# 614 Laser-treatment of Hypertrophic Scar Induces Change to Epidermal Histoarchitecture Correlating to Improved Epidermal Barrier Function

**DOI:** 10.1093/jbcr/irac012.242

**Published:** 2022-03-23

**Authors:** Lesle M Jimenez, Mary A Oliver, John W Keyloun, Taryn E Travis, Jeffrey W Shupp, Lauren T Moffatt, Bonnie C Carney

**Affiliations:** MedStar Health Research Institute, Washington, District of Columbia; Burn Center at MedStar Washington Hospital Center, Washington DC, District of Columbia; Burn Center at MedStar Washington Hospital Center, Washington DC, District of Columbia; MedStar Washington Hospital Center, Washington, District of Columbia; MedStar Washington Hospital Center, Washington DC, District of Columbia; Burn Center at MedStar Washington Hospital Center, Washington DC, District of Columbia; Medstar Health Research Institute, Washington DC, District of Columbia

## Abstract

**Introduction:**

Mechanisms and timing of hypertrophic scar (HTS) improvement with laser therapy are incompletely understood. Epidermal keratinocytes influence HTS through paracrine signaling, yet they are understudied compared to fibroblasts. It was hypothesized that fractional ablative CO_2_ laser scar revision (FLSR) would change the fibrotic histoarchitecture of the epidermis in HTS.

**Methods:**

Duroc pigs (n=4 FLSR and n=4 controls) were injured and allowed to form HTS. HTS and normal skin (NS) were assessed weekly by non-invasive skin probes measuring trans-epidermal water loss (TEWL) and biopsy collection. There were 4 weekly FLSR treatments. Early laser treatment began on day 49, and late began on day 77. Punch biopsies from NS and HTS were processed and stained with H&E. Image J was used to obtain epidermal thickness and rete ridge ratios (RRR). Gene and protein expression of involucrin (IVL) was examined through qRT-PCR and immunofluorescent (IF) staining.

**Results:**

After treatment, peeling sheets of stratum corneum were apparent which were not present in the controls. TEWL was increased in HTS vs. NS at day 49 indicating decreased barrier function (42.2±8.0 vs. 22.0±4.62g/m^2^h, p=0.05). In the early group, TEWL was significantly decreased at week 4 to 16.4±3.5 g/m^2^h (p< 0.05). The late group was not significantly altered from NS at the pre-laser timepoint (day 77=12.1±1.99 g/m^2^h). Hence, there was no decrease in TEWL post-FLSR. After 4 sessions, epidermal thickness was significantly increased in treated scars in both FLSR groups (early:pre=85.6±6.8 vs. week 4=115.2±12.0 µm, p< 0.01) and (late:pre=75.2±6.6 vs. week 4=125.7±12.0 µm p< 0.001, n=8 scars,). There was no increase in controls. Late intervention significantly increased RRR (pre=1.3±0.1 vs. week 4=1.9±0.1, n=8 scars, p< 0.05*),* and early treatment trended towards increase (pre=1.17±0.05 vs. week 4=1.4 + 0.1). There was no increase in controls. There was increased IVL gene expression in HTS vs. NS that decreased after FLSR. Eight scars had up-regulated gene expression of IVL vs. NS levels pre-treatment (FC >1.5) compared to 4 scars at week 4. This was confirmed by IF where IVL staining decreased at week 4.

**Conclusions:**

Changes in epidermal HTS histoarchitecture and expression levels of epidermal differentiation markers were induced by FLSR. The timing of laser intervention contributed to differences in TEWL, epidermal thickness, and RRR.

